# Collision Detection and Identification on Robot Manipulators Based on Vibration Analysis

**DOI:** 10.3390/s19051080

**Published:** 2019-03-03

**Authors:** Feiyan Min, Gao Wang, Ning Liu

**Affiliations:** 1 Department of Electronic Engineering, College of Information Science and Technology, Jinan University, Guangzhou 510632, China; minfeiyan@aliyun.com (F.M.); twangg@jnu.edu.cn (G.W.); 2 Robotics Research Institue of Jinan University, Guangzhou 510632, China

**Keywords:** manipulator, model independent method, collision detection, collision identification, vibration analysis, artificial neural network

## Abstract

Robot manipulators should be able to quickly detect collisions to limit damage due to physical contact. Traditional model-based detection methods in robotics are mainly concentrated on the difference between the estimated and actual applied torque. In this paper, a model independent collision detection method is presented, based on the vibration features generated by collisions. Firstly, the natural frequencies and vibration modal features of the manipulator under collisions are extracted with illustrative examples. Then, a peak frequency based method is developed for the estimation of the vibration modal along the manipulator structure. The vibration modal features are utilized for the construction and training of the artificial neural network for the collision detection task. Furthermore, the proposed networks also generate the location and direction information about contact. The experimental results show the validity of the collision detection and identification scheme, and that it can achieve considerable accuracy.

## 1. Introduction

Industrial robots play an important role in the modern manufacturing industry, and human-friendly robots will soon become flexible and versatile coworkers in the industrial setting [1]. One of the core problems in human–robot interaction is the detection of collisions between robots and the industrial environment, including humans and other manufacturing structures. Indeed, industrial robots should be able to operate in very dynamic, unstructured, and partially unknown environments, sharing the workspace with the human user, and preventing upcoming and undesired collisions [2].

Furthermore, researchers are getting more interested in gathering the maximum amount of information from the impact event, such as the contact position, direction and intensity, in order to let the robot react in the most appropriate fashion. Related concepts include collision avoidance [3], and collision isolation, identification, classification and reaction [2]. Particularly, the concept collision isolation aims at localizing the contact point, or at least which link out of the n-body robot collided. Furthermore, collision identification is to determine the directional information of the generalized collision force [2].

Different approaches for detection of robot collisions have been presented in the literature. A first intuitive approach is to monitor the current transient in robot electric drives, looking for shock changes within currents caused by collisions [4,5,6,7]. A second approach is based on the tactile sensors laying inside robot skins. The more common approach is the so-called model-based method (the state observer or Kalman filter method), and the detection algorithms are mainly based on the evaluation of monitoring signals (motor currents, difference between actual and predicted torques, etc.), which should be below some setting values, otherwise collision alarms are generated.

A major practical problem in these methods is the selection of thresholds for the monitoring signals, since the modeling error and sensor noise affect the monitoring signal in the same way as collision disturbance. A good detection algorithm therefore must distinguish the effect of modeling error and sensor noise on monitoring signal from that of a real collision. For this reason, it usually leads to a tradeoff between sensitivity and false alarm rate, with a risk of excessively conservative threshold [2,5]. To overcome this problem, some different methods are proposed. A dynamic threshold is defined in [4] to represent the residual dependence on the state of the robot (position, velocity, acceleration) using fuzzy logic rules. Furthermore, in [6], authors propose an adaptive detection algorithm based on a state-dependent dynamic threshold. In recent years, some extended state observer [8] and sliding mode observer [9] methods are proposed to obtain more effective detection performance.

Most of these evaluation algorithms mainly concentrate on the time domain information of motor torque deviation (The motor currents can be considered as another form of torque). A considerable alternative is to use frequency domain features for detection purposes.

This paper describes a novel detection method based on vibration modal feature generated by collision. The natural frequency and vibration modal features of collision experiments are extracted for the constructing and training of several structures of Back Propagation (BP) neural network. The research result shows that this method can be used for the detection of collision with considerable accuracy, not only for the detection of collision occurrence, but also for the positioning and direction determination.

A remarkable contribution of this paper is to introduce frequency and vibration information for the detection of robot collision. The vibration information is independent of the dynamic model and can be easily acquired with acceleration sensor, which has greatly developed in recent years. Since the occurrence of collision can happen anywhere along the robot structure, the acceleration sensor is easy to stall and suitable for the detection use. Furthermore, the industrial robots are not usually equipped with torque sensor because of cost and structural constraints, and this research provides an opportunity for MEMS accelerometer.

Accurate analysis of natural frequency and modal shape is fundamental for mechanical design, dynamic identification and control of high-speed manipulator [10,11]. In recent years, the acceleration and vibration analysis method has been gradually used for the status monitoring of manipulator robot. In [12], the authors propose a fault detection method for industrial welding robot. In their study, joint acceleration of robot is considered as evaluation criteria and their evaluation algorithm is based on neural network. The article [13] presents a method for processing and analyzing the measurement signals used in the problem of diagnosing the state of a manipulator’s tool. The analysis algorithm is performed within the time and frequency domain. The signal utilized in the research is the mechanical vibrations and the rotation speed of the tool. In the work [14], frequency domain analysis method is researched for the event classification in robot assisted deburring. Power spectrum density (PSD) of sensor data acquired between certain sample rates is calculated, and it is then used for classifying vibration signal generated by the spindle from the vibration signal acquired during the deburring process. The paper [15] presents a signal fusion method based on accelerometer and encoder in serial robots. Besides, a number of studies have presented the data-based method for mechanical system monitoring based on vibration signals [16,17,18,19,20]. In most of this research, specific forms of artificial neural network are proposed and implemented [21,22,23,24,25].

The rest of this paper is organized as follows. Section 2 describes the frequency domain features and parameters of vibration of manipulator in case of collisions. Section 3 presents the architecture of our detection neural network, together with vibration modal analysis method; Section 4 presents some results and experiments obtained with the proposed method; and finally, Section 5 addresses the main conclusion and future work.

## 2. Vibration Modeling and Feature Extraction

This section address the vibration presentation and its characteristics. First, the modeling method of vibration response is proposed with dynamic equations and transfer functions. Then, the typical features are analyzed based on the mathematical model, together with illustrative examples.

### 2.1. Vibration Response under Collision and Its Mathematical Modeling

In this paper, our research focuses on the vibration response of multiple test points along manipulator structure under several experiments. An effective method for dealing with vibration of kinematic chain mechanism is the elastodynamic modeling and analysis [10,11]. In this method, each critical mechanical structure and component is simplified with stiffness, viscous and mass parameter. As for a n-dof manipulator, it is composed of a series of motors, gears, links and joints, some of which will generate deformation and vibration under collision. For this reason, the dominant vibration structures can be considered as elastic bodies with certain stiffness and viscous coefficients.

We consider a n-dof manipulator with *m* dominant vibration structures. Its axis displacement vector can be defined as:

(1) $$q=\left[\begin{array}{c} q_{D}\\ q_{M} \end{array}\right]\in R^{N}$$

where $q_{D}={\left[q_{1},q_{2},\cdots ,q_{m}\right]}^{T}$ is the vibration deviation, and $q_{M}={\left[q_{m+1},q_{m+2},\cdots ,q_{m+n}\right]}^{T}$ denotes the joint displacements. Furthermore, we assume the equilibrium points of $q_{D}$ is ${\bar{q}}_{D}={\left[{\bar{q}}_{1},\cdots ,{\bar{q}}_{m}\right]}^{T}$. The dynamic equation of manipulator can be written as:

(2) $$M\left(q\right)\ddot{q}+C\left(q,\dot{q}\right)\dot{q}+G\left(q\right)=\left[\begin{array}{c} \tau_{D}\\ \tau_{M}+\tau_{f} \end{array}\right]$$

the variable $\tau_{M}$, $\tau_{f}$ and $\tau_{D}$ denotes the joint torque generated by motor, friction and structure deformation respectively. We denote $\tau_{D}={\left[\tau_{1},\cdots ,\tau_{m}\right]}^{T}$, and $\tau_{M}={\left[\tau_{m+1},\cdots ,\tau_{m+n}\right]}^{T}$. The subscript *D* denotes the dominant vibration structures, and *M* denotes the drive motor of manipulator.

On the other hand, the torque generated by the deformation of vibration structures can be given by:

(3) $$\tau_{D}=K_{p}\left(\bar{q_{D}}-q_{D}\right)-K_{v}q_{D}$$

where $K_{p}=diag\left(k_{p1},k_{p2},\cdots ,k_{pm}\right)$, $K_{v}=diag\left(k_{v1},k_{v2},\cdots ,k_{vm}\right)$ is the vibration dynamic coefficient matrix. Furthermore, $k_{pi}$, $k_{vi}$ denotes the stiffness and vicious coefficient of ith dominant vibration structure respectively.

We assume that the gravity and friction is compensated by feedback control loop, and the Coriolis and centrifugal effect generated by structure deformation is relatively small. In most cases, the displacement of $q_{D}$ is much smaller than $q_{M}$, and the inertia matrix M(q) is mainly determined by joint displacement. Furthermore, the torque vector generated by collision is assumed as $f_{ext}$. Then we get the dynamic function of manipulator as follows

(4) $$\left[\begin{array}{c} 0\\ \tau_{M} \end{array}\right]=M\left(q_{M}\right)\left[\begin{array}{c} {\ddot{q}}_{D}\\ {\ddot{q}}_{M} \end{array}\right]+\left[\begin{array}{cc} K_{v} & C_{D}\left(q_{M},{\dot{q}}_{M}\right)\\ 0 & C_{M}\left(q_{M},{\dot{q}}_{M}\right) \end{array}\right]\left[\begin{array}{c} {\dot{q}}_{D}\\ {\dot{q}}_{M} \end{array}\right]+\left[\begin{array}{c} K_{p}\\ 0 \end{array}\right]\left(q_{D}-\bar{q_{D}}\right)+\left[\begin{array}{c} {J_{D}}^{T}\\ {J_{M}}^{T} \end{array}\right]f_{ext}$$

where ${J_{D}}^{T}$ and ${J_{M}}^{T}$ is the associated geometric contact Jacobian matrix to *m* vibration structures and *n* robot motors, respectively. However, the collision torque vector $f_{ext}$ and contact Jacobian ${J_{D}}^{T}$, ${J_{M}}^{T}$ is typically unknown.

Denoting $x_{1}=\left[\begin{array}{c} {\dot{q}}_{D}\\ q_{D}-\bar{q_{D}} \end{array}\right]$, $y_{1}=q_{D}-\bar{q_{D}}$, and $x_{2}=\left[\begin{array}{c} {\dot{q}}_{M}\\ q_{M} \end{array}\right]$, $y_{2}={\dot{q}}_{M}$, an *n* inputs and m+n outputs state-space equation is obtained

(5) $$\left\{\begin{array}{c} {\dot{x}}_{1}=A_{1}x_{1}+B_{11}y_{2}+B_{12}f_{ext}\\ {\dot{x}}_{2}=A_{2}x_{2}+B_{21}u+B_{22}f_{ext}\\ y_{1}=C_{1}x_{1}\\ y_{2}=C_{2}x_{2} \end{array}\right.$$

where *u* is the active motor torque, and $f_{ext}$ is torque vector generated by collision, and the related parameter matrix is given by:

$$\begin{array}{l} A_{1}=\left[\begin{array}{cc} -{M(q_{M})}^{-1}K_{v} & -{M(q_{M})}^{-1}K_{p}\\ I & 0 \end{array}\right]\\ B_{11}=\left[\begin{array}{c} -{M(q_{M})}^{-1}C_{D}\left(q_{M},{\dot{q}}_{M}\right)\\ 0 \end{array}\right],\;B_{12}=\left[\begin{array}{c} {J_{D}}^{T}\\ 0 \end{array}\right],\;C_{1}={\left[\begin{array}{c} 0\\ I \end{array}\right]}^{T}\\ A_{2}=\left[\begin{array}{cc} -{M(q_{M})}^{-1}C_{M}\left(q_{M},{\dot{q}}_{M}\right) & 0\\ I & 0 \end{array}\right]\\ B_{21}=\left[\begin{array}{c} -{M(q_{M})}^{-1}\\ 0 \end{array}\right],\;B_{22}=\left[\begin{array}{c} {J_{M}}^{T}\\ 0 \end{array}\right],\;C_{2}={\left[\begin{array}{c} I\\ 0 \end{array}\right]}^{T} \end{array}$$

Then we get the modal analyzed transfer function from collision torque $f_{ext}$ to vibration deformation $y_{1}$ as follows

(6) $$P\left(s\right)=\frac{Y_{1}\left(s\right)}{F_{ext}\left(s\right)}=C_{1}{\left(sI-A_{1}\right)}^{-1}\left[B_{12}+B_{11}C_{2}{\left(sI-A_{2}\right)}^{-1}B_{22}\right]$$

We considered the structure of system matrix $A_{1}$ and $A_{2}$, and we got that $rank(A_{1})=2m$ and $rank(A_{2})=n$. Let $\lambda_{Di},{\bar{\lambda }}_{Di}\left(i=1,2,\cdots ,m\right)$ be 2m eigenvalues of $A_{1}$, and $\lambda_{0}\left(=0\right)$, $\lambda_{Mi}\left(i=1,2,\cdots ,n\right)$ be the n+1 eigenvalues of $A_{2}$.

From the partial fraction expansion, the modal analyzed transfer function P(s) becomes

(7) $$P\left(s\right)=\sum_{k=1}^{m}\frac{\Phi_{k}}{s^{2}+2\xi_{k}\omega_{Dk}s+{\omega_{Dk}}^{2}}+P_{1}\left(s\right)$$

By modal analysis, P(s) can be expressed as a linear sum of m+n vibration modes, *m* modes of which are generated from the *m* dominant vibration structures. Furthermore, the other *n* poles which come from $A_{2}$, correspond to the *n*-dof active dynamic of manipulator.

Then the natural frequency of vibration along manipulator can be obtained by $\omega_{Di}=\left|\lambda_{Di}\right|\left(i=1,2,\cdots ,m\right)$ and $\omega_{Mj}=\left|\lambda_{Mj}\right|\left(j=1,2,\cdots ,n\right)$.

The matrix $\Phi_{k}$ corresponds to the rigid body vibration mode and is positive semidefinite, and it has the following structure

(8) $$\Phi_{\cdot \cdot i}=\left[\begin{array}{cccc} \phi_{11i} & \phi_{12i} & \cdots & \phi_{1ni}\\ \phi_{21i} & \phi_{22i} & \cdots & \phi_{2ni}\\ \cdots & \cdots & \cdots & \cdots \\ \phi_{l1i} & \phi_{l2i} & \cdots & \phi_{lni} \end{array}\right]$$

For any $\phi_{kji}\in \Phi_{\cdot \cdot i}$, the subscript k∈(1,2,⋯,l) denotes the serial number of vibration test position, j∈(1,2,⋯,n) denotes the serial number of collision torque in vector $f_{ext}$, and i∈(1,2,⋯,m) is the number of vibration modes.

We considered the structure of system matrix $A_{1}=\left[\begin{array}{cc} -{M(q_{M})}^{-1}K_{v} & -{M(q_{M})}^{-1}K_{p}\\ I & 0 \end{array}\right]$, it is mainly determined by inertia matrix $M(q_{M})$, vibration dynamic coefficient matrix $K_{p}$ and $K_{v}$, of which the latter two remain nearly unchanged. That means the natural frequencies $\omega_{Di}\left(i=1,2,\cdots ,m\right)$ will vary with joint displacement $q_{M}$.

As the stiffness coefficients $k_{pi}\left(i=1,2,\cdots ,m\right)$ of manipulator structure are usually considerably large, there exist several eigenvalues of $A_{1}$ relatively larger than that of $A_{2}$, which comes from the active dynamic of robot and reflects the normal working frequency of manipulator. For this reason, there exist several natural frequencies of vibration that are obviously bigger than the frequency band of active dynamic of robot, i.e., $\exists \omega_{Di}\left(i=1,2,\cdots ,m\right)$, and $\omega_{Di}>\omega_{Mj}\left(j=1,2,\cdots ,n\right)$ hold.

So we get an important conclusion: the dominant natural frequency of vibration under collisions is independent of the dynamic property of robot. When the inertia matrix is given, the natural frequency under collision should remain the same during different dynamic processes or static statuses.

Furthermore, the modal matrix $\Phi_{k}$ depends on the value of geometric contact Jacobian matrix ${J_{D}}^{T}$ and ${J_{M}}^{T}$. For this reason, the contact position information can be induced by the value of vibration mode matrix $\Phi_{k}$.

### 2.2. Vibration Features Analysis and Illustrative Example

**Figure 1:**
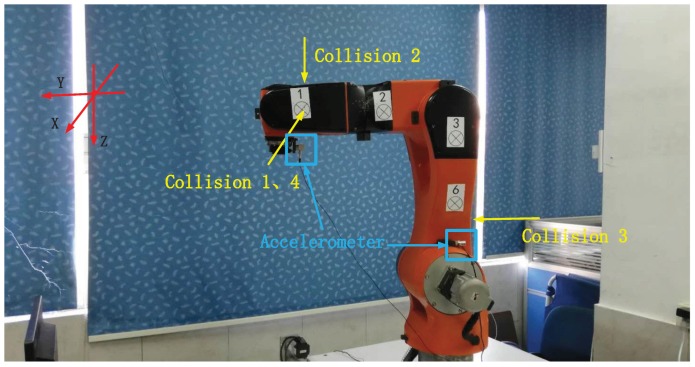
Experiment setup.

This section illustrates the characteristics of vibration modal shape of manipulator under collisions with example. The test data were collected on the STR6-05 robot arm (see Figure 1), a 6-DOF heavy-load manipulator. Four consecutive collision experiments were conducted while the end-effector moved in line; the collision conditions are listed in Table 1.

**Figure 2:**
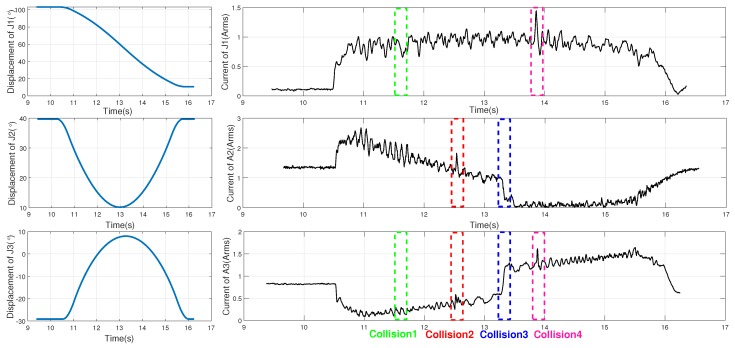
Joint displacement and current during the experiment.

The joint displacement and motor current signals of joint 1, 2 and 3 are shown in Figure 2. We label the contact time of corresponding collision events on the figure. We find that there are some shock changes in the motor currents but little change in joint displacements. However, it is hard to detect collision events from current signals directly because of dynamic change and the noise involved in signals, particular for heavy-load manipulator. As shown in the figure, we cannot find some obvious features for collision 1.

For vibration signal measuring, the 1A113E and 1A114A industrial accelerometer (made by Donghua Testing Technology) with NI-9232 data acquisition module is used. The 1A113E accelerometer (uniaxial) is mounted beside joint 2, perpendicular to the direction of joint 1 and joint 2. The 1A114A accelerometer (triaxial) is equipped beside the end-effector of manipulator.

**Figure 3:**
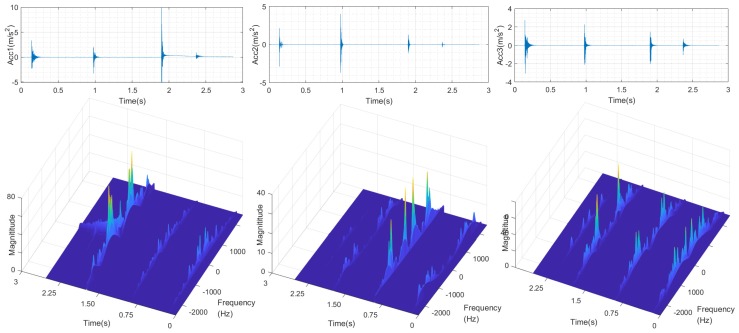
Vibration modes in experiment.

The vibration acceleration and vibration modal is shown in Figure 3. The first signal comes from 1A113A located beside joint 2, and the latter comes from 1A114A beside end-effector, corresponding to two perpendicular directions.

The frequency characteristic of each accelerometer within sliding windows is shown in Figure 3. There are four obvious frequency charts corresponding to four collisions in each vibration signal. The peak frequencies, i.e., the natural frequencies of three accelerations corresponding to one collision are approximately the same, with different energy densities. The magnitudes of all the peak frequencies constitute the modal matrix Φ in Equation (8).

Figure 3 shows that the vibration modes of different parts along robot structure have the following characteristics:

1. The contact position information can be analyzed with vibration modes of different sensors. The main vibration frequency of nearer sensors is usually higher than sensors far from contact position. That is because manipulator structure can be seen as a low-pass filter, the higher frequency vibration signal is reduced during the propagation process. For example, the magnitude of the 3rd collision in the 1st sensor is comparatively higher than that in the 2nd sensor signals. That is because the 3rd collision is conducted near the base, which is much nearer to the 1st signal.
2. Similarly, the contact direction can also be determined with vibration modes. The relative energy density value of different directions in the triaxial accelerometer can be used to detect the contact direction. Generally speaking, the vibration of collision direction may have the comparatively higher energy density. For example, the 1st and 2nd collision took place at the same part with different directions, the 2nd magnitude is higher than the 1st one in the 2nd signal while the 2nd magnitude is smaller than the 1st one in the 3rd signal, as is shown in the figure.
3. Furthermore, contact material information can also be reflected by vibration modal. The band width of the 4th collision is much lower than the other 3. That is because the frequency band of human hand contact force is comparatively narrow.

**Figure 4:**
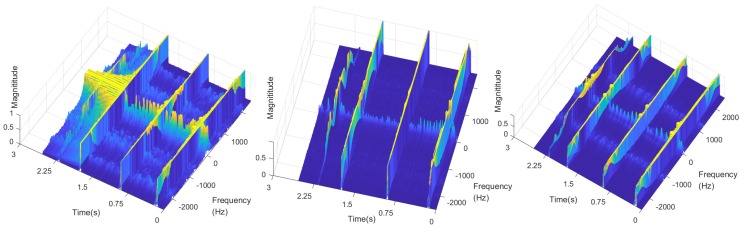
Low magnitude segment of vibration modes in experiment.

Limiting the range of magnitude below 1 dB, we got the frequency characteristic of normal dynamic comparative to collisions in Figure 4. It shows that the natural frequency of active dynamic appears at low frequency segment (always below 50 Hz), while the vibration frequency by collision appears at intermediate segment. That means the eigenvalues of $A_{2}$ is much smaller than that of $A_{1}$ in Equation (5), and the dynamics of normal operation do not affect the natural frequencies of collisions.

Obviously, the natural frequencies and modal is mainly dependent on the inertia matrix, and independent of the robot dynamic. This means that the detection algorithm can be designed without considering the dynamic property of the robot, and the training and test samples for the model independent method can be collected in some simple or static scenarios.

Since collision will generally cause shock vibration, and its natural frequencies are mainly contained in the high-frequency domain, several symptom parameters in the frequency domain can be selected to represent the collision event. Extracting collision information from frequency domain signal requires proper understanding of the process. For frequency features, such as natural frequencies, the vibration modal shown in the spectrum often has direct or indirect connection to certain dynamic events.

## 3. Collision Detection and Identification Method Based on Vibration Features

Based on the vibration features discussed above, we proposed a learning-based algorithm for the detection, isolation and identification of collisions.

**Figure 5:**
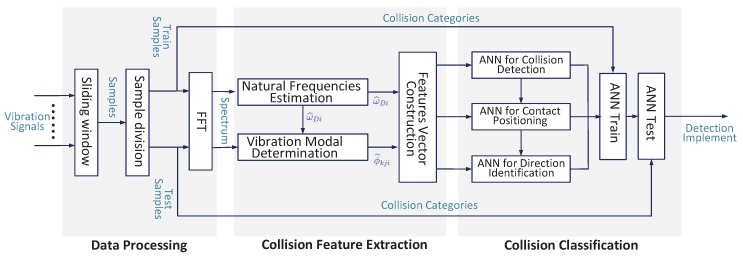
Vibration signal based detection framework.

The proposed method mainly contains three parts: vibration mode analysis, collision detection, and collision identification. As shown in Figure 5, the vibration signals are first recorded by accelerometer sensors. The vibration mode related features are then extracted from the vibration signals. Any change in the vibration mode from normal condition can indicate the occurrence of collision. Three different Back Propagation neural networks are developed for the detection of collisions (BP1), the isolation of contact part (BP2), and identification of direction (BP3). BP2 and BP3 should be activated only if some collisions are detected by BP1, and the input of BP3 varies with the output of BP2.

### 3.1. Vibration Mode Estimation

The main objective of vibration mode estimation is to determine the main natural frequencies $\omega_{Di}\left(i=1,2,\cdots ,m\right)$, and the values of vibration mode corresponding to each natural frequency $\phi_{kji}\in \Phi_{\cdot \cdot i}$, where *k* denotes the serial number of sensors, *j* denotes the serial number of collision force, and i∈(1,2,⋯,m) denotes the number of vibration modes.

In the collision experiment, the result of measurement is strings of acceleration values in discrete moments. The acceleration signal should be properly processed in order to build the collision classifier. For the discrete string a1(k), a2(k), and a3(k), fast Fourier transform (FFT) is used to determine vibration spectrum within a sliding window which has lower computational cost and better accuracy performance. In our research, the sampling rate is 3.2 k Hz, and the width of sliding window is 320 samples with 50% overlap. Furthermore, the cycle time of detection algorithm is 0.1 s.

For the spectrum of each sampling window, we proposed a peak frequency-based method to determine the natural frequency ${\hat{\omega }}_{Di}\left(i=1,2,\cdots ,m\right)$, the estimation of $\omega_{Di}$. For each, the proposed approach consists of the following steps:

*Step 1: For acceleration signal k∈(1,2,⋯,l), set a tolerance error *Δ* and δ for spectrum analysis;*

*Step 2: Find all the local maximum power and local minimum power from start frequency $f_{0}$ to cut-off frequency $f_{e}$, such that there is no other power value larger than current maximum value between the two adjacent local minimum powers, and there is no other power value less than current minimum value between the two adjacent local minimum power densities; The difference between adjacent maximum and minimum power density should be larger than *Δ*;*

*Step 3: Collect all the local maximum power values and corresponding frequencies;*

*Step 4: Calculate Num, the number of local maximum power density;*

*Step 5: If Num>m, then Δ=Δ−δ, repeat step 2 to step 4;*

*Step 6: Record current local maximum power values $P_{ki}\left(i=1,2,\cdots ,m\right)$ and corresponding frequencies $f_{ki}\left(i=1,2,\cdots ,m\right)$;*

*Step 7: For other acceleration signal k∈(1,2,⋯,l), repeat step 1 to step 6*;

*Step 8: Rank all the frequency values $f_{ki}\left(k=1,2,\cdots ,l;i=1,2,\cdots ,m\right)$, and divide them into m groups with maximum intervals.*

*Step 9: Calculate the average frequencies within each group, these values are the estimation of modal frequency, ${\hat{\omega }}_{Di}\left(i=1,2,\cdots ,m\right)$.*

**Figure 6:**
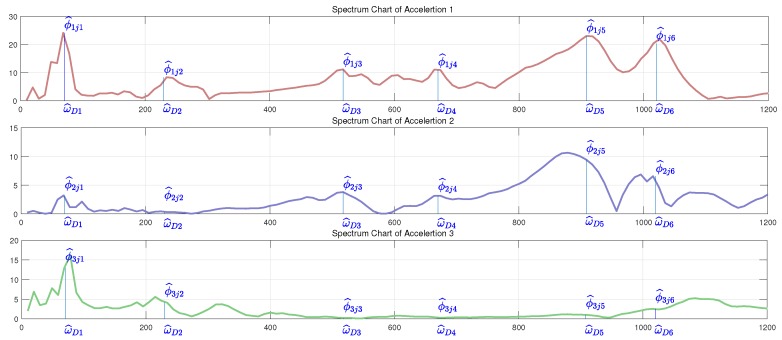
Joint displacement and current during the experiment.

With the estimation of modal frequencies, the vibration modal matrix $\hat{\Phi }$ can be determined by extracting the magnitude of corresponding frequency in the spectrum chart, as it shows in Figure 6. In this figure, we get 6 natural frequencies ${\hat{\omega }}_{D1},{\hat{\omega }}_{D2},\cdots ,{\hat{\omega }}_{D6}$ from the measurements of three acceleration signals in collision experiment *j*. The magnitude values at each characteristic frequency are the estimation of vibration modal. Obviously, there is little error of estimation, e.g., the estimation ${\hat{\phi }}_{2j5}$. However, this error has limited influence on the final detection result because this detection is based on the synthesis of multiple vibration modal. In this way, we get the estimation of vibration modal matrix of experiment *j*:

(9) $${\hat{\Phi }}_{\cdot j\cdot }=\left[\begin{array}{cccc} {\hat{\phi }}_{1j1} & {\hat{\phi }}_{1j2} & \cdots & {\hat{\phi }}_{1j6}\\ {\hat{\phi }}_{2j1} & {\hat{\phi }}_{2j2} & \cdots & {\hat{\phi }}_{2j6}\\ {\hat{\phi }}_{3j1} & {\hat{\phi }}_{3j2} & \cdots & {\hat{\phi }}_{3j3} \end{array}\right]$$

### 3.2. Proposed Artificial Neural Network

**Figure 7:**
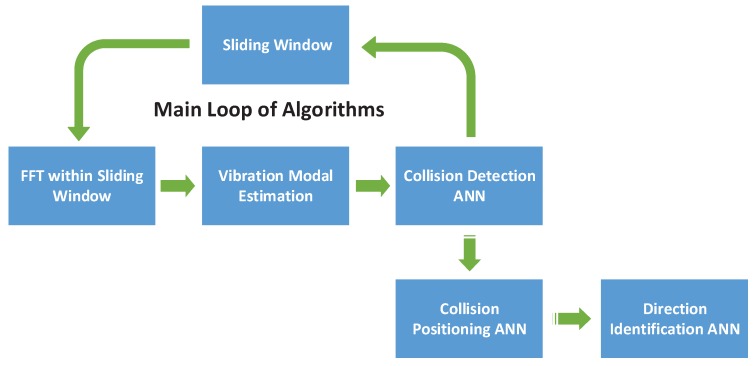
Procedure of detection algorithm.

In our method, 3 BP networks are implemented for the collision detection, positioning and direction identification respectively. The collision detection artificial neural network together with the modal analysis algorithm should be executed within each sliding window. Once a collision event is detected, the collision positioning artificial neural network is launched with the current vibration modal data. In addition, the input of the 3rd network is dependent on the output of collision position information. The operation process of relevant algorithms is displayed in Figure 7.

**Figure 8:**
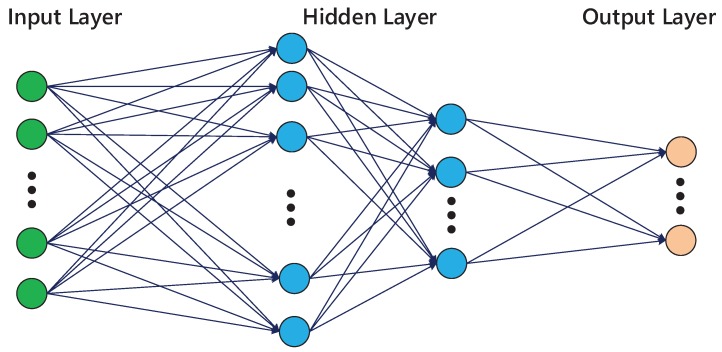
Back Propagation (BP) artificial neural network structure.

B-P ANN consists of an input layer, hidden layers, and an output layer of neurons. A neuron serves as a processing unit in which output is a linear or nonlinear transformation of its inputs. The neurons, as a group, serve to map the input vibration modal features to the desired collision patterns. The structure of BP network is shown in Figure 8.

The output signal of hidden layer and output layer can be described in the following equations:

(10) $$m_{j}\left(t\right)=f\left(\sum_{j=1}^{10}w_{ij}\left(t\right)t+b_{j}\right)$$

where $m_{j}\left(t\right)$ is the output of current neurons, $w_{ij}$ is the weight of the connection between current layer neurons and its input layer neurons, $b_{j}$ is the bias of the jth neuron of current layer. The activation function of output layer is linear function, while the hidden layer uses sigmoid function

(11) $$f\left(t\right)=\frac{1}{1+e^{-t}}$$

Considering the characteristic of vibration modes discussed in Section 2, we selected the most important features for each kind of detection task, as shown in Table 2, Table 3 and Table 4.

As the natural frequencies are mainly dependent on inertia matrix $M_{qM}$, we selected the displacement of joint 2 and joint 3 as input features of detection. The displacements of other joints have little influence on inertia matrix.

The contact position and direction of collision can affect the comparative vibration magnitude of sensor on different positions and directions. We select relative modal between test position for the positioning of contact, and relative modal between test direction beside contact position for direction identification.

## 4. Experiment and Discussion

### 4.1. Experiment Dataset Preparation and Training Procedure

**Figure 9:**
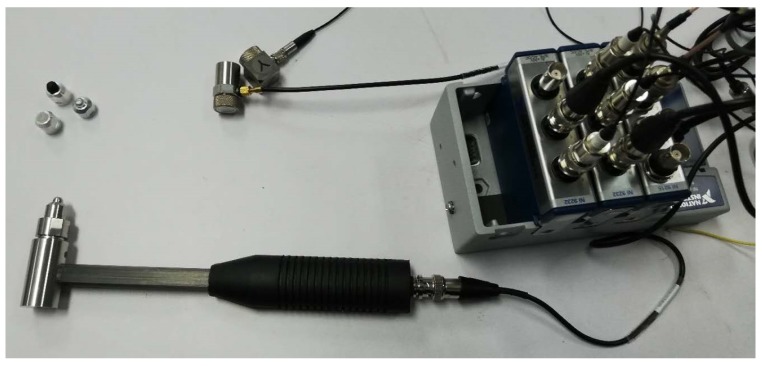
Data acquisition module for collision experiment.

The experiment dataset is collected on the platform in Figure 1. The robot is controlled with PD control law during the experiment. An uniaxial accelerometer is mounted beside joint 2, perpendicular to the direction of joint 1 and joint 2. In addition, a triaxial accelerometer is equipped beside the end-effector of manipulator. All acceleration data is recorded by NI-9232 data acquisition module. Our experiments are conducted with an aluminum impact hammer, which is also connected to NI-9232 data acquisition module. In this way, the collision time and force data is recorded, as shown in Figure 9.

**Figure 10:**
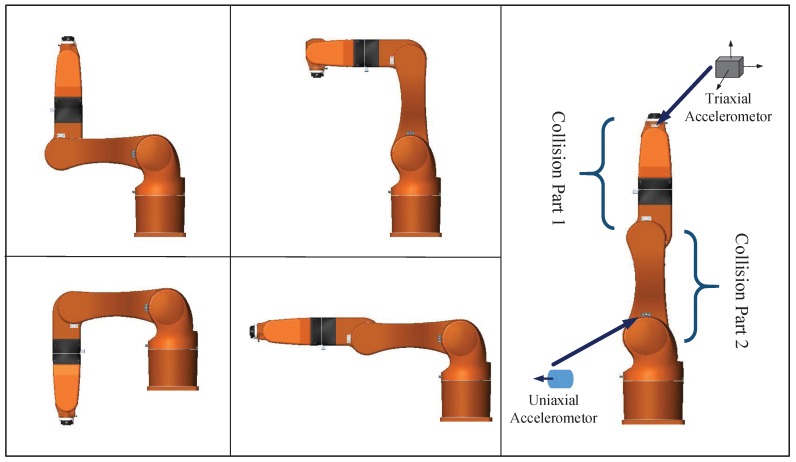
Five testing patterns of manipulator.

The collision experiments are performed with eight selected contact points, with different direction and force intensity. As the natural frequencies of vibration are mainly dependent on inertial matrix and joint displacement (see Section 2.1), we select five typical working patterns (see Figure 10) as testing standard. Our collision experiments are performed while the robot is transforming randomly from one pattern to another.

Hundreds of collision experiments are performed on the platform. The vibration modal data of acceleration signals during these experiments are analyzed with the sliding-window method introduced above. Typical vibration modal data sets with corresponding experiment conditions are collected for classification research. Meanwhile, we also collect the vibration modal data during collision-free operations. We get in total 800 experiment samples, of which 50 percent are collision samples performed on 8 different contact positions along robot structure. Eighty-five percent of these samples are selected randomly for training and the remaining are used for testing.

### 4.2. Detection and Identification Results

In this subsection, several test divisions of experiment data are utilized to evaluate the efficiency of the proposed method. Our BP-ANN algorithms are taken from the Neural Network toolbox in Matlab. At the training stage, we optimize the weights and bias parameters by minimizing mean squared error, according to Levenberg-Marquardt optimization.

For the detection of collisions, BP networks with different architecture are constructed and trained. Table 5 lists the accuracy of detection ANN of different architectures (The structure i−j−k−o means a network with *i* input neurons and *o* output neurons, and *j* and *k* stands for the number of neurons in hidden layers ). It is clear to see from the table that the proposed method has considerable accuracy for collision detection, and an average accuracy of 0.95 can be obtained with 1 or 2 hidden layers.

All the vibration modal data of actual collision are used for the training and testing of positioning neural networks. Considering the geometric layout of the STR6-05 robot, the moveable structure is divided as two parts, i.e., one part is link 3, and the other contains link 4, link 5 and link 6, as shown in Figure 10. Table 6 lists the accuracies of different layers of positioning networks. In addition, the BP networks with 2 hidden layers are suitable for the positioning task.

As the vibration modal features of collision direction are mainly reflected by the acceleration signals nearby the collision point, the input of direction identification network is determined by the output of positioning network. Table 7 lists the accuracy of network of 3 architectures. The training and testing samples come from the vibration data of collisions on link 4, link 5 and link 6, which is near to the acceleration sensors on end-effector. It is obvious that the BP network with 2 hidden layers can obtain comparative stabilization accuracy.

Considering all the results comprehensively, the proposed method can be utilised for the detection, positioning and identification of collisions with considerable accuracy. By analyzing, we find that the positioning error and identification error is mainly derived from boundary samples, that is the collisions near joint 3. One more accelerometer located beside joint 3 may be used for the enhancement of accuracy.

By analysing the training and test procedure, we can find that an experiment of 300 collision samples is enough for the artificial network training for any 6−dof manipulator with different sensor placement scheme, and it can be accomplished in half or one hour with well designed scenarios and test procedures.

### 4.3. Rapid Prototyping System Design

In order to realize the online test of the proposed method, the computation complexity and detection time of the related algorithms should be estimated. As discussed above, the main calculation consumption includes three parts, namely, the fast Fourier transform of vibration acceleration data, the estimation of natural frequency and modal, and the node outputs update of neural network.

**Figure 11:**
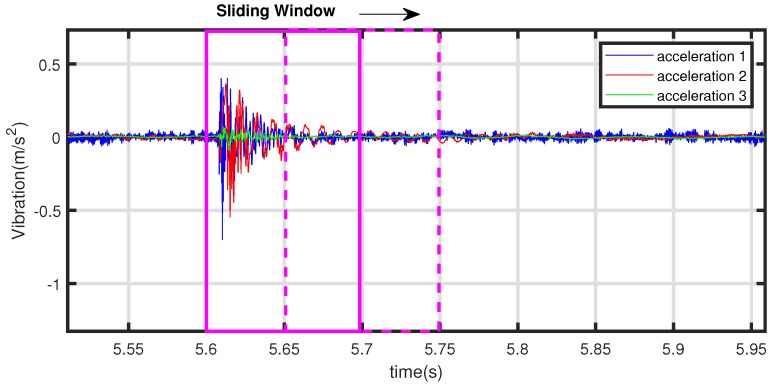
Sliding window for online vibration detection test.

It can be seen from Section 2.2 that the collision vibration frequency of the robot is mainly between 50 Hz and 1500 Hz, and we set the sampling rate to 3200 samples/second. On the other hand, in order to ensure the spectral characteristics have a sufficient resolution, we select a sliding window of 0.1 s for each cycle, and the number of sampling points N=320 for Fourier transform, as shown in Figure 11. The overlap amount of the sliding window is 50 percent, that is, the calculation cycle of the proposed algorithm is 0.05 s. Within each computing period, Butterfly fast Fourier transform is adopted, and the total number of real multiplications required by FFT of *N* points is $2N*log_{2}\left(N\right)$, and the total number of real numbers added is $2N*log_{2}\left(N\right)$ for each vibration signal.

For the natural frequency and modal estimation algorithm, the main computational cost of the algorithm is the sorting algorithm of spectrum amplitude, and the computational complexity of the algorithm is $O(N*log_{2}\left(N\right))$ [26], that means, the computational cost of the frequency and modal estimation algorithm can be ignored relative to the fast Fourier transform.

For the status update of the neural network, each neuron includes multiple addition operations, multiplication operations and exponential operations, and the exponential operation can be converted into a number of addition and multiplication operations. For the collision detection neural network with typical structure of 27-6-4-1, the addition calculation times of a single update is 278, and the total number of multiplication is 98. For the positioning and direction identification neural network with one or two hidden layers, the calculation cost is of the same magnitude.

Therefore, the main computational cost of this method is derived from the fast Fourier transform. In addition, within a single update cycle (50 ms), the addition and multiplication times of the algorithm is about 22,000 and 21,000 respectively. The total computation consumption of this method is about the same size as the traditional state observer method with Newton-Euler Function, as discussed in [27].

**Figure 12:**
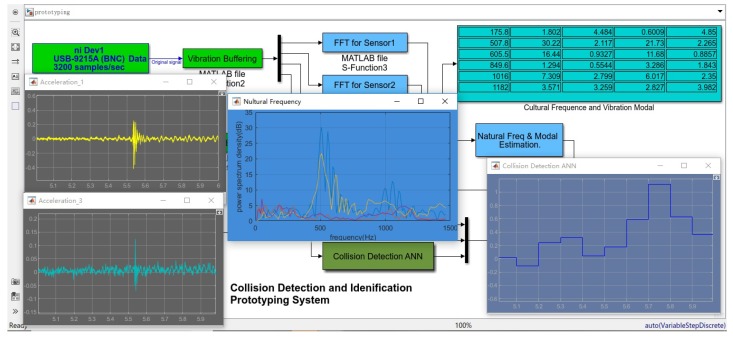
A Rapid Prototyping System.

Based on the above analysis, we use Simulink/Data Acquisition Toolbox to develop a rapid prototyping system, as shown in Figure 12. The vibration data within each sliding window is buffered, and then used for FFT, modal estimated, and neural network status updates. The system is a slower-than-real-time system due to data caching and operating system. It shows that the collision detection time is about 0.1 s, and the isolation and identification time will be a little longer.

## 5. Conclusions and Discussion

In this work, we present a model independent method for robot collision detection, positioning and identification. The vibration signals are analyzed and used for the construction and training of BP neural network. The test results of the experiment confirm that it is possible to build a monitoring algorithm with considerable accuracy. The conclusions may be summarized as follows:

1. With a small amount of training samples (about 300–500 samples), the proposed method can provide considerably high accuracy, and it can be conducted in half an hour or one hour with any kind of manipulators. Therefore, this method has the potential to be implemented in real application scenarios.
2. The detection and identification method is mainly dependent on the frequency domain features of collision, the time-domain features can also be added to improve detection accuracy and computational efficiency in further research.
3. The bandwidth of collisions on heavy load manipulator is mainly below 1500 Hz, and the bandwidth of light robot should be much less than that value. This means some high-performance MEMS accelerometers may be utilised on some occasions.
4. The main calculation consumption of the proposed method comes from the FFT of vibration signal, some dedicated FFT chips can be utilized to improve detection performance.

## Figures and Tables

**Table 1:** Experiment conditions for vibration modal test.

Experiment Index	Contact Position	Contact Direction	Contact Material
1	Near end-effector	X	Aluminum impact hammer
2	Near end-effector	Z	Aluminum impact hammer
3	Near base	Y	Aluminum impact hammer
4	Near end-effector	X	Human hand

**Table 2:** The features used for the detection of collisions.

Features	Function Equation
Geometric Appearance	$q_{M2},q_{M3}$
vibration Frequencies	${\hat{\omega }}_{Di}\;\left(i=1,2,\cdots ,m\right)$
vibration modal	${\hat{\phi }}_{kji}\;\left(k=1,2,\cdots ,l;j=1,2,\cdots ,n;i=1,2,\cdots ,m\right)$

**Table 3:** The features used for the isolation of contact position.

Features	Function Equation
Geometric Appearance	$q_{M2},q_{M3}$
Vibration Frequencies	${\hat{\omega }}_{Di}\;\left(i=1,2,\cdots ,m\right)$
Vibration modal of end-effector	${\hat{\phi }}_{1ji},\;{\hat{\phi }}_{1ji}\left(i=1,2,\cdots ,m\right)$
Relative modal between test position	${\hat{\phi }}_{k,j,i}/{\hat{\phi }}_{k-1,j,i}\;\left(k=2,3,\cdots ,k;i=1,2,\cdots ,m\right)$

**Table 4:** The features used for the identification of collision direction.

Features	Function Equation
Geometric Appearance	$q_{M2},q_{M3}$
Vibration Frequencies	${\hat{\omega }}_{Di}\;\left(i=1,2,\cdots ,m\right)$
Relative modal between test direction of contact position	${\hat{\phi }}_{cx,j,i}/{\hat{\phi }}_{cy,j,i},\;{\hat{\phi }}_{cx,j,i}/{\hat{\phi }}_{cz,j,i},\left(i=1,2,\cdots ,m\right)$

**Table 5:** Accuracy of collision detection.

Network Architecture	Actual Status	Number of Samples	Detection Result		
			Collision	Non-Collision	Accuracy
27-5-1	Collision	53	51	2	0.962
	Non-collision	59	3	56	0.949
27-10-1	Collision	53	51	2	0.962
	Non-collision	59	4	55	0.932
27-6-4-1	Collision	53	50	3	0.943
	Non-collision	59	3	56	0.946

**Table 6:** Accuracy of collision positioning.

Network Architecture	Actual Position	Number of Samples	Positioning Result		
			Part 1	Part 2	Accuracy
27-10-1	Part 1	23	18	5	0.783
	Part 2	31	5	26	0.838
27-5-5-1	Part 1	23	20	3	0.870
	Part 2	31	4	27	0.871
27-10-5-1	Part 1	23	21	2	0.913
	Part 2	31	4	27	0.871
27-15-10-3-1	Part 1	23	20	3	0.870
	Part 2	31	2	29	0.936

**Table 7:** Accuracy of direction identification.

Network Architecture	Actual Direction	Number of Samples	Identification Result			
			X-Direction	Z-Direction	Y-Direction	Accuracy
33-8-2	X-direction	18	14	3	1	0.778
	Z-direction	10	3	7	0	0.700
	Y-direction	3	1	0	2	0.667
33-10-6-2	X-direction	18	15	2	1	0.833
	Z-direction	10	1	9	0	0.900
	Y-direction	3	1	0	2	0.667
33-15-10-2	X-direction	18	15	3	0	0.833
	Z-direction	10	2	8	0	0.800
	Y-direction	3	0	0	3	1.000
